# Variety of Culturable Bacteria Associated with Subclinical Mastitis in Dairy Cows, Based on the Simpson’s and Shannon–Wiener Diversity Indices

**DOI:** 10.3390/antibiotics15070683

**Published:** 2026-07-12

**Authors:** Michael Farre, Lærke Boye Astrup

**Affiliations:** SEGES Innovation, Agro Food Park 15, 8200 Aarhus, Denmark; lbas@seges.dk

**Keywords:** subclinical mastitis, SCC, diversity index, dairy cow

## Abstract

**Background:** Subclinical mastitis is a major challenge in dairy production, contributing to antimicrobial use, production losses, and persistent infection pressure while often remaining undetected due to the absence of clinical signs. Using somatic cell count (SCC) data to distinguish newly infected from chronically infected cows is epidemiologically important, yet little is known about how infection dynamics relate to bacterial diversity in these groups. The present study aimed (i) to quantify the herd-level association between newly and chronically subclinically infected cows based on SCC, and (ii) to compare the abundance distribution of culturable bacteria in these groups using ecological diversity indices. Methods: We combined longitudinal Dairy Herd Improvement (DHI) data from 88 Danish dairy herds with quarter-level microbiological culture and MALDI-TOF MS identification from 1738 cows. **Results:** A strong positive herd-level correlation was observed between newly and chronically infected cows (Spearman’s *r* = 0.76, *p* < 0.001), indicating substantial shared variation without implying causality. Both Simpson (D) and Shannon–Wiener (H′) indices differed significantly between newly and chronically infected cows, demonstrating distinct bacterial abundance distributions. Non-aureus staphylococci dominated both groups, whereas *Staphylococcus aureus* and *Streptococcus uberis* were markedly more prevalent in chronically infected cows. Conclusion: the findings show that newly and chronically subclinical mastitis represent distinct epidemiological and ecological states, underscoring the need to combine SCC data with species-specific diagnostics to support effective herd-level mastitis control.

## 1. Introduction

Mastitis in dairy cows is the most important disease for the dairy sector worldwide, causing economic losses, concerns regarding antimicrobial usage, and severe animal welfare issues [1]. Recent global estimates indicate that clinical mastitis accounts for approximately USD 13 billion in annual losses, with subclinical mastitis contributing an additional USD 9 billion [2].

Simulation studies under Danish conditions have demonstrated that intervention strategies can influence both herd profitability and mastitis incidence [3]. Also, if we focus on herd level, mastitis represents a major source of economic loss at the herd level [4]. Also, authors such as [5,6,7] have demonstrated substantial economic consequences associated with elevated mastitis prevalence and increased somatic cell counts, addressing the impact on economic performance.

SCC in milk primarily reflects immune system cells [8]. Therefore, several studies use the SCC to distinguish healthy cows from cows with an inflammatory response in the udder, where the cut-off between healthy and inflamed is often set to SCC ≥ 200,000 cells/mL milk [9,10]. As such, the SCC is also used to identify cows with a subclinical inflammatory response in the udder, namely cows with an elevated SCC ≥ 200,000 cells/mL milk but no clinical symptoms. If the SCC continues to be above 200,000 cells/mL, it further defines the cow as chronically infected, clinically or subclinically [11]. Distinguishing newly infected from chronically infected cows is epidemiologically important because these categories capture different aspects of udder health dynamics. New infections are informative of current transmission pressure and ongoing failures of prevention, whereas chronic infections reflect persistence over time and contribute disproportionately to the long-term burden of subclinical mastitis detected through SCC-based surveillance. This distinction is also relevant for management, because chronic cases often have lower bacteriological cure rates and may require different treatment, segregation, or culling decisions than newly infected cows. Accordingly, separating new from chronic subclinical mastitis is essential for interpreting herd udder health data and for designing effective control strategies [8,10,11].

Compared with clinical mastitis, subclinical mastitis lacks obvious clinical signs and therefore often remains undetected and untreated for prolonged periods. This contributes to a higher number of affected cows and allows infections to persist without triggering relevant biosecurity actions at both cow and herd levels [12]. Moreover, several studies have shown that subclinical mastitis is related to a somewhat different array of bacteria than clinical mastitis [10,12,13]. Recent studies show that subclinical mastitis is a polymicrobial disease, making it even more difficult to manage [10,13,14]. These recent studies focus on the udder microbiome as an entity rather than on udder infections caused by intruding pure cultures. Their results raise a new awareness of how to understand and target mastitis.

As such, the microbiome perspective on udder health shifts our understanding of mastitis from a simple infection toward a condition characterized by microbial dysbiosis of the mammary gland. To improve management of subclinical mastitis, we therefore need more studies that focus on the composition of bacteria in the udder.

Improving udder health management of subclinical mastitis is essential, as it reduces milk losses, improves milk quality, enhances animal welfare, and supports overall herd productivity. Subclinical mastitis is one of the most prevalent diseases affecting dairy cattle worldwide. Recent meta-analyses estimate a cow-level prevalence of approximately 42–46%, indicating that nearly one out of every two dairy cows may be affected by subclinical intramammary inflammation [15,16]. Several factors influence the occurrence of mastitis, including cow-related factors such as parity and production level, as well as pathogen-related factors including the type and epidemiology of the causative organism [17,18,19,20].

Mastitis prevention, such as hygiene, milking procedures, and environmental management, emphasizes to reduce new intramammary infections, and systematic monitoring to reduce new intramammary infections is advised by the National Mastitis Council (NMC) [21].

Monitoring udder health relies primarily on indicators such as SCC and bacteriological culture. When used in combination, these data sources provide complementary information that can support earlier detection of udder health problems and facilitate more targeted management decisions at the herd level.

We therefore set up the present study with two objectives: The first objective was to analyze the correlation between the number of newly and chronically infected cows as determined by SCC. The second objective was to describe and compare the abundance distribution of culturable bacteria in new and chronic subclinical mastitis via the Simpson and Shannon–Wiener indexes, respectively [22,23].

## 2. Materials and Methods

### 2.1. Microbiological Examination of Quarter Milk Samples

Quarter milk samples were examined at the Centre for Diagnostics, Technical University of Denmark as described earlier by Svennesen et al. 2023 [24]. In brief, samples were analyzed within a maximum of 6 weeks from sampling. Milk samples were thawed and homogenized, 10 µL from each sample was plated on blood agar with 5% calf blood (SSI Diagnostica A/S, Hillerød, Denmark) and incubated at 37 °C and read after 24 and 48 h. For culture-negative samples at 48 h, the milk sample was re-cultured and incubated at 37 °C in CO_2_ and read after 24 and 48 h. If there was still no growth, the sample was considered culture negative.

We defined culture-positive samples as those with at least one colony, corresponding to a cut-off of 100 CFU/mL of milk. We discarded samples that showed growth of three or more morphologically different colony types as visually contaminated. Samples with one or two morphologically different colony types had a representative colony of each kind sub-cultivated and read after 24 h to obtain pure cultures for identification and freezing of every bacterial isolate. If the sub-culture still appeared as morphologically combined it was sub-cultured and incubated for 24 h again. Irrespective of if one or 2 sub-culture steps were conducted to obtain pure cultures, the resulting pure cultures were identified in triplicate by Matrix-assisted laser desorption-ionization Time-of-Flight (MALDI-TOF MS) (Microflex LT/SH, Bruker Daltonik^®^ GmbH, Bremen, Germany). In the identification process, we considered a MALDI-TOF MS score ≥ 2.00 as a correct identification at the species level; we accepted scores between 1.70 and 1.99 at the genus level, and we regarded scores ≤ 1.69 as no identification, labeling the pathogen as “not identified” according to the guidelines by Bizzini et al. 2010 [25]. Moreover, MALDI-results were only considered valid if all triplicate identifications provided the same bacteria and within the same score-interval of either ≥2.00, between 1.70 and 1.99 or ≤1.69. We discarded samples contaminated on MALDI-level, if the sample contained more than two different pathogens at the species level following MALDI-TOF MS analysis. We excluded both visually contaminated samples and samples identified as contaminated after MALDI-TOF MS analysis from the data analysis as “contaminated”. We further categorized the included samples as pure or mixed cultures (two different species) based on the MALDI-TOF MS analysis only. This way, the bacterial identification both disregarded if cultures appeared as visually combined at the first culture and double checked for contamination in samples that seemed pure cultures. Thereby, the bacterial identification allowed colonies to be morphologically affected by the presence of milk constituents and/or neighbor colonies without this leading to assumptions or selection of colonies. Hence, in all samples that were not visually contaminated, the types of colonies were treated as equally important, and with the same level of identification reproducibility.

### 2.2. Data Analysis

The BTSCC 12-month geometric mean, median, and quartiles, as well as the herd 12-month average new infection risk, mean, median, and quartiles, were calculated as illustrated in Table 1, to present the variation in BTSCC and new infection risk in the dataset.

The initial data analyses were conducted in R (Version 4.3.3, ‘Angel Food Cake 2024,’ https://www.R-project.org/ accessed on 15 August 2024) and *p* ≤ 0.05 was deemed statistically significant.

In this study we initially made descriptive statistics for BTSCC and new infection risk and then applied seven analyses to the data from DHI recordings and the microbiological data. As the first analysis, we tested the DHI data for normal distribution of cows defined as infected and non-infected, based on a SCC cut-off at >200,000 cells/mL.

Second, we analyzed the level of correlation between the total numbers of chronically and newly infected cows, based on DHI recordings, with Spearman’s rank correlation test [26].

Then, in our third and fourth analyses, we calculated the Simpson and Shannon–Wiener index analysis, respectively, at the herd level, where we used the definitions from DHI SCC to define newly and chronically infected cows combined with microbiological data from the cow’s quarter samples, according to the equations below:


$$Simpson\;Index\left(D\right)=\sum_{i=1}^{N}({p_{i})}^{2}$$

$$Simpson\;Index\left(H\right)=\sum_{i=1}^{S}p_{i}{\ln p}_{i}$$

The Simpson index considers the number of microbiological species, providing insights into the dominant species. In the Simpson index, *i* represents each individual species, *p_i_* is the proportion of samples belonging to species *i* (calculated as *n_i_* divided by *N*), *n_i_* is the number of individuals in species *i*, and *N* is the total number of individuals across all species. For the Shannon–Wiener index, *S* is the total number of species. The Shannon–Wiener index considers richness and evenness.

Based on our microbiological definitions, we could have up to eight pathogens included from each single cow: maximum contribution from 4 quarters with a mixed bacterial sample (two bacterial species) in each quarter.

In our fifth and sixth analyses, we calculated the index for newly infected and chronically infected cows between the herds, both for the Simpson and the Shannon–Wiener indices, using the herd level indices as input for the analysis. To quantify the variation in diversity between herds for both the Simpson and Shannon–Wiener indices, we calculated the mean, median, standard deviation, and 95% confidence intervals by determining the percentiles of the bootstrap-estimated values (2.5% and 97.5%).

Lastly, as the seventh analysis, we used Wilcoxon tests to compare the Simpson and Shannon–Wiener indices for new infection and chronically infected cows pairwise between the herds, because we wanted to identify if there were any differences between herds.

In addition to the seven analyses, the bacterial culture data was assessed and is presented descriptively.

## 3. Results

We identified a total of 498 dairy herds that met the inclusion criteria. Of these, 88 herds were enrolled based on randomization, which comprised 18% of the eligible herds in our study. The enrolled herds ranged from 105 to 1291 dairy cows, with an average of 326 dairy cows. The BTSCC 12-month geometric mean, median, and quartiles, as well as the herd 12-month average new infection risk, mean, median, and quartiles, were calculated for the 88 herds as illustrated in Table 1, to present the variation in BTSCC and new infection risk in the dataset.

We reduced the number of herds in the calculation of both indexes due to unmet microbiological criteria. That is, cows who fulfilled the criteria on SCC but not for the microbiology of any of their respective quarter milk samples due to, e.g., contaminated samples, which in some herds lead to all cows of a group being omitted. Hence, the newly infected group included 74 dairy herds, and the chronically infected group included 82 dairy herds for microbiology, respectively. In total, we enrolled 1738 dairy cows in the study, distributed to 755 newly infected cows and 904 chronically infected cows.

At the quarter level, the total number of samples was 6713, out of which 2387 samples were excluded. Hence, we included 4326 samples distributed as 1851 from newly infected cows and 2475 from chronically infected cows, respectively. The quarters excluded from the dataset were culture-negative, contaminated, no-ID from the MALDI-TOF MS analysis, or missing samples (Figure S1). The number of quarter milk samples included in the study per herd was in the range (3–69) of newly infected cows and the range (4–74) samples in chronically infected cows.

### 3.1. Spearman’s Rank Correlation Between Chronically and Newly Infected Cows

**Figure 1:**
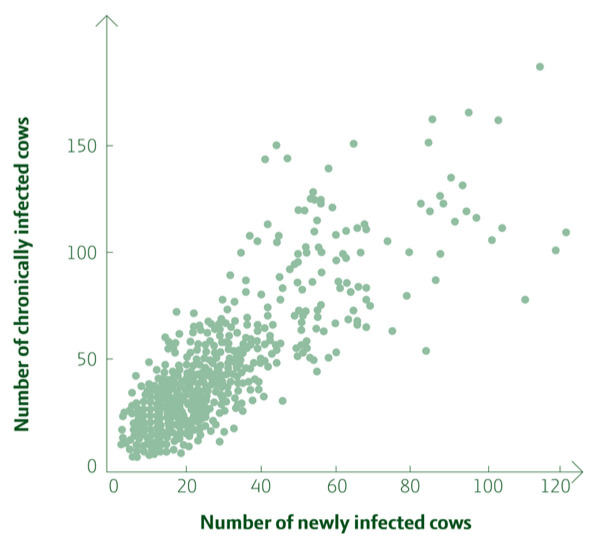
The correlation between the total numbers of chronically and newly infected cows in the 88 dairy herds, as determined by DHI recordings using a cut-off of 200,000 cells/mL. Each herd has 12 months of data illustrated in Figure 1.

Figure 1 illustrates the correlation between chronically and newly infected cows. Spearman’s rank correlation at the herd level for the correlation coefficient (r) between chronically infected and newly infected cows was *r* = 0.76 (*p* < 0.001).

This result shows a strong positive correlation (*r* = 0.762). The coefficient of determination (*r*^2^ = 0.58) indicates substantial shared variation between newly and chronically infected cows, though this association does not imply causation.

This underlines that herds with a higher number of chronically infected cows also tend to have a higher number of newly infected cows identified from SCC data. However, this association does not indicate directionality. Newly infected cows may progress to chronic infection over time, and both groups likely reflect the same underlying infection pressure and control challenges within the herd.

### 3.2. Simpson Diversity Index

We calculated the Simpson diversity index for each herd and then compared the index between the herds.

**Figure 2:**
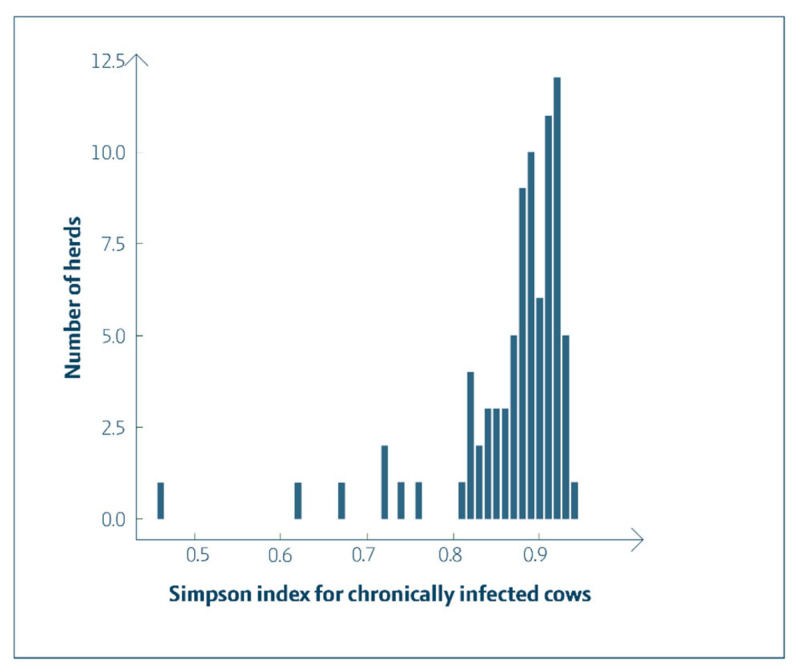
The interval of the Simpson index between the herds in chronically infected cows.

**Figure 3:**
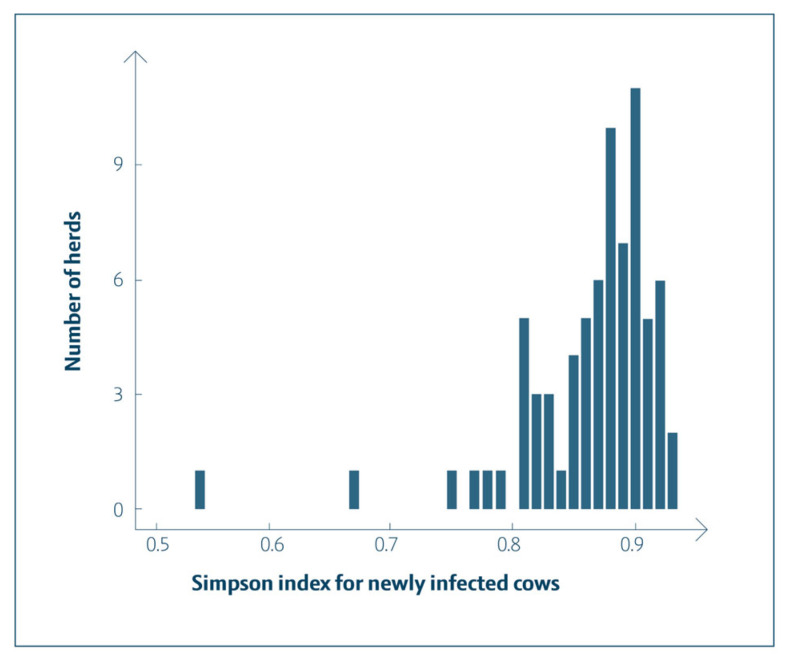
The interval of the Simpson index between the herds in newly infected cows.

Between the herds, we found that the newly infected cows with a positive, non-contaminated bacterial culture had an interval of *D* = (0.53–0.93). For chronically infected cows, we found an interval of *D* = (0.46–0.94) between the herds. Hence, we found a broader interval in chronically infected cows than in newly infected cows. The Simpson index quantifies the probability that two bacteria randomly selected from a sample will belong to the same bacterial species [26]. In addition, the index gives a single value that reflects the diversity of bacterial species, facilitating comparisons of different environments in terms of groups of cows or dairy herds. In the present dataset, the samples represent a SCC at the cow level, combined with a positive microbiological culture at the quarter level. The calculated Simpson indexes for chronically and newly infected cows are shown in Figure 2 and Figure 3. The *y*-axis represents the number of quarter samples for each herd, while the *x*-axis displays the corresponding Simpson index values. The right-skewed distribution of the Simpson index values for both chronically and newly infected cows suggests that microbial communities in many herds were dominated by a limited number of species.

Additionally, we analyzed the standard deviation, mean, median, and confidence intervals to explore the data, using the output from the Simpson index in Table 2.

Because the number of samples from the two groups of cows varied considerably between farms, we compared the number of samples with the Simpson diversity index for all farms with an index value below *D* = 0.8 to assess if low index value was correlated to low sample number. This comparison showed that in farms with a Simpson diversity index value below *D* = 0.8, the number of samples from each herd varied in the newly infected group from (3–44) samples and in the chronically infected group from (5–103) samples.

The Wilcoxon test showed a significant difference between indexes of newly and chronically infected cows within each herd *W* = 3.185 (*p* = 0.004). This suggests that the two groups of subclinical mastitis cows have statistically different values for the Simpson diversity index.

### 3.3. Shannon–Wiener Index

**Figure 4:**
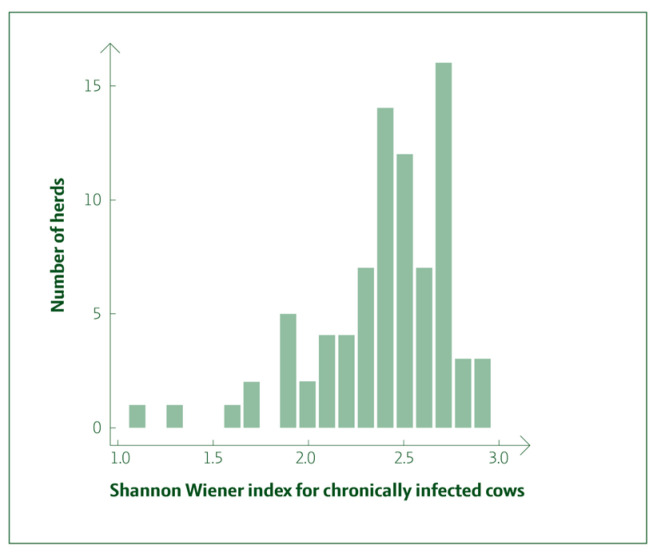
The interval of the Shannon–Wiener index for chronically infected cows. The *y*-axis shows the number of quarter samples for each herd, and the *x*-axis shows the index.

**Figure 5:**
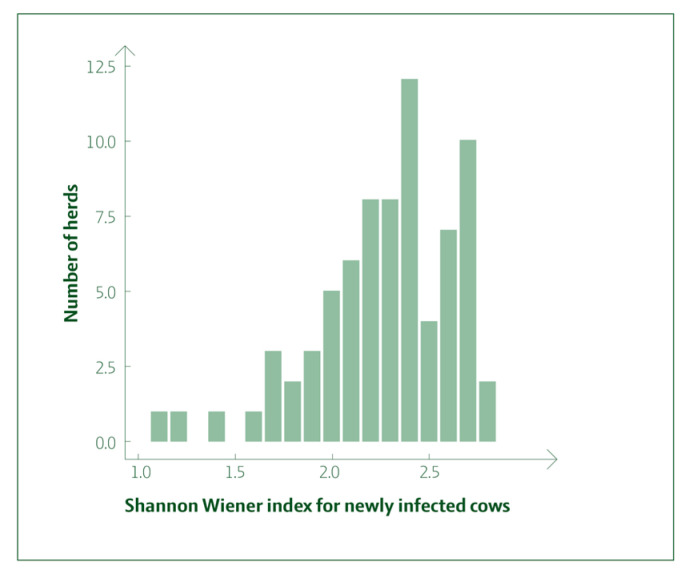
The interval of the Shannon–Wiener index for newly infected cows. The *y*-axis shows the number of quarter samples for each herd, and the *x*-axis shows the index.

We calculated the Shannon–Wiener index for each herd and then compared the indices between the herds. We calculated the Shannon–Wiener index for cows defined as newly infected for each herd with an interval of *H*’ = (1.09–2.77) between the herds. We calculated the index for cows defined as chronically infected in each herd, which showed an interval of *H*′ = (1.13–2.91) between the herds. Figure 4 and Figure 5 illustrate the calculated Shannon–Wiener index for chronically and newly infected cows. The right-skewed distribution of the Shannon–Wiener index values for both chronically and newly infected cows suggests that microbial communities in many herds were dominated by a limited number of species.

Additionally, we calculated the standard deviations, mean, median, and confidence intervals to further strengthen the analysis, using the output from the Shannon–Wiener index in Table 3.

Because the number of samples from the two groups of cows varied considerably between farms, we compared the number of samples with the Shannon–Wiener index for all farms with an index value below *H*′ = (2.0) to assess if low index value was correlated to low sample number. This comparison showed that in farms with a Shannon–Wiener index value below 2.0, the number of samples from each herd varied in the newly infected group from (3–13) samples and in the chronically infected group from (5–41) samples. We used the Wilcoxon test to compare the indices calculated for newly and chronically infected between the herds, which resulted in *W* = 3.795 (*p* = 0.007). This suggests that the two groups of subclinical mastitis cows have statistically different values for the Shannon–Wiener index.

### 3.4. Microbiological Culture

Microbiological culture results are shown in Table 4 and Table 5. The results are based on a systematic approach to microbiological identification with clearly defined procedures for standard culture followed by up to two sub-culture steps and a final systematic MALDI-TOF protocol. The protocols entail clear and systematic decision-making on contamination and standardized detection levels, respectively. The protocol was developed by the author at the Centre for Diagnostics, Technical University of Denmark in 2019 to ensure that all samples were treated systematically and to avoid hierarchical pre-selection of cultures within samples. Accordingly, the results in Table 4 and Table 5 can be used as reference for other studies on subclinical mastitis, because they include all identified bacteria at the species level, without grouping and without bias from unstructured pre-treatment and/or hierarchical selection of reported species. We identified 139 different bacterial species in samples from chronically infected cows and 119 in samples from newly infected cows. In both the chronically and the newly infected cows, the predominant bacteria were non-aureus staphylococci and mammaliicocci (*NASM)* as a group, 1201 and 1008, respectively. Also, both chronically and newly infected cows had *S. epidermidis* as the most prevalent species, with 316 and 220 samples positive for this species, respectively. In contrast, *S. aureus* was the second most prevalent species in chronically infected cows, n=289, whereas *S. aureus* was only the seventh most prevalent species in the newly infected cows, n = 100. Likewise, *S. uberis* was the third most prevalent species in the chronically infected cows, n = 176, but the tenth most prevalent species in the newly infected cows (n = 74). In both groups of cows, Gram-negative bacteria were scarce, with only 60 and 54 samples containing Gram-negative bacteria in chronic and newly infected cows, respectively. In the chronically infected cows, *E. coli* was the predominant Gram-negative bacteria, present in 21 out of 60 Gram-negative samples. In the newly infected cows, *Psychrobacter* spp. and *Acinetobacter* spp. were the dominant and almost equally prevalent Gram-negative bacteria, present in 17 and 11 out of 54 Gram-negative samples, respectively.

## 4. Discussion

This study addresses two objectives: (i) to analyze the correlation between the number of chronically infected and newly infected cows as determined by SCC, and (ii) to describe and compare the abundance distribution of culturable bacteria in newly and chronically subclinical mastitis cows using ecological diversity indices.

In this study, we combined longitudinal DHI data with snapshot but extensive quarter-level microbiological culture to investigate the relationship between chronic and new intramammary infections and to characterize bacterial diversity in subclinical mastitis at the herd and between-herd levels. Our results demonstrate a strong epidemiological association between chronic and newly infections and reveal differences in bacterial diversity and pathogen composition between newly and chronically infected cows.

We identified a strong positive correlation between the number of chronically and newly infected cows at the herd level with Spearman’s correlation test *r* = 0.76, (*p* < 0.001), indicating that approximately 58% of the variance in the number of newly infected cows is statistically associated with variance in the number of chronically infected cows. However, this association does not imply directionality or causation, as newly infected cows may subsequently progress to chronic infection, and both measures likely reflect shared underlying infection pressure within the herd.

This finding underscores the relevance of chronically infected cows in herd-level mastitis dynamics and supports the continued applicability of established mastitis control principles, such as those outlined in the NMC 10-point plan, where identification and appropriate management of chronically infected cows remain central components. However, the observation that dominant bacterial organisms differ between newly infected and chronically infected cases indicates that chronic cows should not be regarded as direct reservoirs of the same pathogens causing new infections. Rather, the association likely reflects persistent infection pressure and stage-specific infection dynamics within the herd [1,8,9]. Our results further emphasize the value of systematic DHI data analysis for herd veterinarians and dairy farmers to identify infection patterns and target herd-specific udder health interventions. Thus, the first objective of the study was met by demonstrating a strong, statistically significant association between SCC-defined chronic and new intramammary infections at the herd level.

While SCC data is essential for identifying infection dynamics, our findings clearly demonstrate that SCC alone is insufficient for optimal decision-making. Species-specific diagnoses are essential, as the etiology of infection shows immense variation between individual cows and differs substantially between newly and chronically infected groups of cows. Notably, *Staphylococcus aureus* and *Streptococcus uberis* were considerably more prevalent in chronically infected cows than in newly infected cows, underscoring their importance as persistent pathogens capable of maintaining long-term infection pressure within herds [10,13]. If diagnostic efforts focus solely on clinically affected animals, chronically infected subclinical cows harboring such pathogens may remain undetected, thereby sustaining the risk of new infections. Similarly, management decisions related to, e.g., selective dry cow therapy and culling, may be compromised if all bacteria detected in newly infected cows are treated as true pathogens, since some of them are likely components of the normal udder microbiota rather than invasive infections.

Across both infection groups, NASM constituted the most prevalent bacterial group, consistent with previous reports identifying NASM as the dominant cause of subclinical mastitis in contemporary dairy herds [11,12,14]. *Staphylococcus epidermidis* was the single most prevalent species in both newly infected; with 220 positive and chronically infected cows, we found 316 positive samples, reflecting its strong adaptation to the bovine udder. In contrast, *S. aureus* ranked as the second most prevalent species in chronically infected cows, with 289 positives, but only the seventh most prevalent in newly infected cows, with 100 positive samples. Our data thereby not only supports the association of *S. aureus* with persistence and chronicity as described in clinical mastitis cases but even indicates that the group of subclinical mastitis cases should be considered with more detailed stratification of cows to understand the dynamics of udder health.

In total, we identified 119 different bacterial species in newly infected cows and 139 species in chronically infected cows, illustrating the remarkable microbial complexity of subclinical mastitis. This high species richness challenges traditional classifications of mastitis pathogens into “major” and “minor” categories and highlights the limitations of both classical microbiological diagnostics and interpretations when confronted with such complex datasets. This challenge was further compounded by culture-positive samples without species-level identification using MALDI-TOF MS; we identified 180 in newly infected cows and 209 in chronically infected cows, as well as a substantial number of culture-negative samples, at 1761 in total. Together, these findings underscore the need for alternative analytical approaches capable of capturing and interpreting microbial complexity beyond “major and minor” mastitis pathogens, and even beyond culturable bacteria.

Table 4 and Table 5 provide a structured overview of culturable bacterial species richness in subclinical mastitis samples and may serve as a practical reference framework for future studies. By systematically presenting the distribution and occurrence of species across infection status, these tables can support both research and diagnostic interpretation. The protocol applied was developed within the study to be reproducible, simple, and systematic. All bacterial identifications rely on stringent methodology rather than assumptions. For instance, pre-treatment steps like selection of cultures for sub-culturing is systematic and non-hierarchical, thereby leaving out any bias related to lab-personnel choosing and/or grouping some presumed species over others. This provides the study with a detailed and objective dataset that allows for direct comparison and reproducibility.

Yet, the detection limit of 100 CFU per mL could be challenged if a more detailed view on low-grade bacterial presence must be further investigated. Also, the present protocols rely on standard culture of 48 h on blood agar. This provides simplicity and puts equal weight on all bacteria since no groups of bacteria are provided with improved culture condition over others, such as those that could have been created by the application of special growth media. Yet, the blood agar itself is selecting some species over others. Hence, the protocol inherently favors bacteria suited for growth on blood agar.

The Simpson and Shannon–Wiener indices were applied as quantitative measures of bacterial abundance distribution, capturing both dominance and evenness of culturable species within herds. Between the herds, the Simpson diversity index ranged from *D* = (0.53–0.93) in newly infected cows and *D* = (0.46–0.94) in chronically infected cows, indicating a broader diversity interval in chronically infected herds. However, the number of samples obtained from newly and chronically infected cows varied substantially between herds, which could influence the robustness and comparability of diversity estimates. The Simpson index quantifies the probability that two bacteria randomly selected from a sample will belong to the same bacterial species [23]. Hence, it could be speculated that herds providing few samples would not obtain an index reflecting their true biodiversity. However, our data showed that a low index was not related to a low number of samples as the herds with index < *D* = (0.8) had a range of samples from (3–44) samples in the newly infected group and from (5–103) samples in the chronically infected group. Also, we found a statistically significant difference between herds with *W* = 3.185 (*p* = 0.004).

The Shannon–Wiener index provided complementary insights into microbial diversity. At the herd level, the index ranged from *H*′ = (1.09–2.77) in newly infected cows and *H′* = (1.13–2.91) in chronically infected cows. Again, there was no correlation between samples per herd and index value as in the herds with index < *H*′ = (2.0); the sample range was from (3–13) samples per herd in the newly infected group and from (5–41) samples per herd in the chronically infected group. Similarly, we identified a statistically significant difference between herds, *W* = 3.795 (*p* = 0.007). Nevertheless, we emphasize that disparities in sample numbers may affect the precision of diversity estimates and the likelihood of detecting rare variants, particularly in herds with low sample numbers. Consequently, the observed patterns in both the Simpson and Shannon–Wiener indies should be interpreted with caution, as differences between groups may partly reflect unequal sampling intensity rather than true biological variation. Future studies would benefit from more balanced sampling across herds to improve comparability and strengthen inferences regarding infection dynamics.

Altogether, the right-skewed distribution of both Simpson index values and Shannon–Wiener index values for both chronically and newly infected cows suggests that microbial communities in many herds were dominated by a limited number of species. Hence, not only do the indices point to differences between groups of subclinical mastitis, but the indices also point to the need for accurate diagnostics on individual subclinical cows for proper management decisions, as the microbiology of subclinical mastitis certainly seems too intricate for subclinical cows to be considered as a uniform disease–group and as herds definitely possess their individual microbiological profiles.

Gram-negative bacteria were detected at low frequencies in both infection groups. In chronically infected cows, *Escherichia coli* was the predominant Gram-negative species, with 21 positive samples, whereas *Psychrobacter* spp. had 17 and *Acinetobacter* spp. 11 positive samples, both belonging to the order of Pseudomonadales, dominated among newly infected cows. This distinction may reflect differences between pathogenic Gram-negative infections associated with inflammation and opportunistic or commensal bacteria associated with healthy milk microbiota, although the role of *Acinetobacter* is subject to some dispute [27,28].

These observations further support the notion that bacteria isolated from milk do not necessarily represent pathogenic infections requiring intervention. Instead, at least some findings may reflect normal or transient components of the udder microbiota, underlining the need for further studies focusing on bacterial diversity and community structure in healthy udder.

Beyond the interpretation of the present dataset, the findings may have important applications for both future research and herd-level diagnostics. The extensive species inventories presented in Table 4 and Table 5 provide a detailed overview of culturable bacteria associated with subclinical mastitis and may serve as a useful reference framework for future studies investigating bacterial diversity in dairy herds. Furthermore, the observed differences in bacterial abundance distributions between newly and chronically infected cows suggest that ecological diversity indices may represent a valuable complementary tool for characterizing infection dynamics and microbial community structure. Combining SCC-based classification with species-specific microbiological information may therefore contribute to more targeted herd-level management, improved interpretation of diagnostic results, and more refined mastitis control strategies.

Several limitations must be considered in the present study. The number of herds included in microbiological analyses decreased from 88 to 74 in newly infected cows and to 82 herds in chronically infected cows due to sampling and culture criteria, potentially influencing diversity estimates. However, the prevalence of culture-negative samples (Figure S1) was below the prevalence reported in former studies on subclinical mastitis, indicating that our dataset is not biased by an unusual discharge of samples [29]. In addition, our reliance on culture-based microbiology limits comparability with omics-based studies and likely underestimates the contribution of anaerobic and/or fastidious bacteria known to be part of the udder microbiome [10,13]. However, we aimed to diminish the limitations of culture-based diagnostics by applying systematic MALDI-TOF MS identification of pure sub-cultures of all bacteria in all non-contaminated samples. This way we ensured detailed and objective microbiological data, avoiding any bias caused by subjective assessments based on CFU and/or bacterial species present. Furthermore, the SCC-based classification of chronic infection required only two consecutive elevated recordings, which may have resulted in some overlap between infection groups. Despite these limitations, microbiological differences between groups were still evident, suggesting that the observed patterns are robust.

Accordingly, the second objective was fulfilled by demonstrating statistically significant differences in the abundance distribution of indies between newly and chronically infected cows.

## 5. Conclusions

This study identified a strong association between SCC-defined newly and chronically infected cows at the herd level according to Spearman’s rank correlation, indicating that both groups are linked to the overall infection pressure within dairy herds. In addition, newly and chronically infected cows differed in their bacterial abundance distributions and pathogen composition, suggesting that they represent distinct epidemiological states.

The diversity indices applied in this study provided a useful framework for describing bacterial abundance distributions in the population and highlighted the considerable microbiological complexity of subclinical mastitis. However, the findings should be interpreted considering the study design, including variation in sample numbers between herds and the use of culture-based microbiology.

Overall, the results emphasize that subclinical mastitis cannot be regarded as a uniform condition. Effective herd-level mastitis control is therefore likely to benefit from combining SCC-based monitoring with species-specific microbiological diagnostics to support more targeted and informed management decisions.

## Figures and Tables

**Table 1:** Descriptive statistics for BTSCC and new infection risk in enrolled herds.

	Mean	Median	Min.	Q1	Q2	Q3	Max.
BTSCC cells/mL ^1^	207,000	204,000	89,000	177,000	204,000	237,000	362,000
New infection risk ^2^	11	10	5	8	11	16	20

^1^ Based on processor data, 12 months before the date of the herd’s visit. ^2^ Herd average new infection risk in % for the 12 months before the date of the herd visit, based on DHI data.

**Table 2:** Standard deviation, mean, median, and confidence interval for the Simpson index.

	Mean	Median	SD	95% Confidence Interval
Chronically infected cows	0.87	0.89	0.07	(0.8710–0.8804)
Newly infected cows	0.87	0.88	0.06	(0.8598–0.8804)

**Table 3:** Standard deviation, mean, median, and confidence interval for the Shannon–Wiener index.

	Mean	Median	SD	95% Confidence Interval
Chronically infected	2.4	2.45	0.34	(2.335–2.463)
Newly infected	2.27	2.31	0.37	(2.180–2.331)

**Table 4:** Prevalence and overview of species identified in chronically infected quarters.

Microbiological Organism Chronically Infected	# Quarters		# Quarters		# Quarters
*Staphylococcus epidermidis* ^1^	316	*Staphylococcus succinus* ^1^	5	*Bacillus thermoamylovorans*	1
*Staphylococcus aureus*	289	*Staphylococcus warneri* ^1^	5	*Brachybacterium* sp.	1
*Staphylococcus* sp. ^1^	275	*Bacillus pumilus*	4	*Candida kefyr*	1
No_ID	209	*Chryseobacterium oranimense* ^2^	4	*Cellulomonas* sp.	1
*Streptococcus uberis*	176	*Glutamicibacter* sp.	4	*Corynebacterium casei*	1
*Corynebacterium bovis*	168	*Lactococcus* sp.	4	*Corynebacterium coyleae*	1
*Staphylococcus simulans* ^1^	168	*Streptococcus lutetiensis*	4	*Corynebacterium flaccumfaciens*	1
*Staphylococcus haemolyticus* ^1^	125	*Weissella paramesenteroides*	4	*Corynebacterium imitans*	1
*Staphylococcus chromogenes* ^1^	124	*Brevibacterium* sp.	3	*Corynebacterium riegelii*	1
*Micrococcus luteus*	120	*Candida palmioleophila*	3	*Dietzia* sp.	1
*Corynebacterium amycolatum*	101	*Candida tropicalis*	3	*Enterobacter cloacae* ^2^	1
*Aerococcus viridans*	92	*Citrobacter koseri* ^2^	3	*Enterococcus durans*	1
*Corynebacterium* sp.	89	*Corynebacterium confusum*	3	*Enterococcus gallinarum*	1
*Enterococcus faecium*	83	*Enterococcus cecorum*	3	*Enterococcus hirae*	1
*Lactococcus garvieae*	56	*Klebsiella pneumoniae* ^2^	3	*Enterococcus italicus*	1
*Staphylococcus equorum* ^1^	52	*Paenibacillus amylolyticus*	3	*Enterococcus pseudoavium*	1
*Streptococcus dysgalactiae*	47	*Paenibacillus lactis*	3	*Enterococcus thailandicus*	1
*Staphylococcus hominis* ^1^	40	*Staphylococcus auricularis* ^1^	3	*Exiguobacterium aurantiacum*	1
*Enterococcus faecalis*	27	*Staphylococcus hyicus* ^1^	3	*Glutamicibacter mysorens*	1
*Aerococcus* sp.	26	*Staphylococcus piscifermentans* ^1^	3	*Janibacter indicus*	1
*Enterococcus saccharolyticus*	24	*Staphylococcus saprophyticus* ^1^	3	*Klebsiella aerogenes* ^2^	1
*Corynebacterium frankenforstense*	22	*Streptococcus hyovaginalis*	3	*Kocuria carniphila*	1
*Escherichia coli* ^2^	21	*Streptococcus oralis*	3	*Lactobacillus murinus*	1
*Mammaliicoccus sciuri* ^1^	21	*Streptococcus parauberis*	3	*Lactobacillus* sp.	1
*Corynebacterium xerosis*	17	*Trueperella pyogenes*	3	*Listeria monocytogenes*	1
*Staphylococcus capitis* ^1^	17	*Weissella* sp.	3	*Macrococcus canis*	1
*Staphylococcus rosti* ^1^	17	*Bacillus altitudinis*	2	*Macrococcus* sp.	1
*Staphylococcus xylosus* ^1^	17	*Bacillus simplex*	2	*Micrococcus* sp.	1
*Streptococcus canis*	17	*Bacillus subtilis*	2	*Paenibacillus* sp.	1
*Streptococcus* sp.	17	*Bacillus* sp.	2	*Paenibacillus timonensis*	1
*Candida krusei*	15	*Brevibacterium luteolum*	2	*Paracoccus yeei* ^2^	1
*Lactococcus lactis*	15	*Candida albicans*	2	*Proteus mirabilis* ^2^	1
*Streptococcus agalactiae*	13	*Corynebacterium glutamicum*	2	*Pseudomonas aeruginosa* ^2^	1
*Bacillus* sp.	12	*Globicatella sanguinis*	2	*Pseudomonas* sp. ^2^	1
*Corynebacterium stationis*	10	*Glutamicibacter arilaitensis*	2	*Psychrobacillus psychrotolerans*	1
*Streptococcus dysgalactiae*	10	*Kocuria* sp.	2	*Psychrobacillus* sp.	1
*Psychrobacter pasteurii* ^2^	8	*Moraxella* sp. ^2^	2	*Raoultella ornithinolytica* ^2^	1
*Streptococcus gallolyticus*	7	*Pseudarthrobacter* sp.	2	*Raoultella terrigena* ^2^	1
*Streptococcus pluranimalium*	7	*Rothia* sp.	2	*Serratia marcescens* ^2^	1
*Candida rugosa*	6	*Staphylococcus lugdunensis* ^1^	2	*Serratia* sp. ^2^	1
*Candida* sp.	6	*Staphylococcus vitulinus* ^1^	2	*Sphingobacterium multivorum* ^2^	1
*Enterococcus* sp.	6	*Streptococcus mitis*	2	*Staphylococcus gallinarum* ^1^	1
*Acinetobacter* sp. ^2^	5	*Acinetobacter junii* ^2^	1	*Staphylococcus muscae* ^1^	1
*Corynebacterium aurimucosum*	5	*Arthrobacter gandavensis*	1	*Staphylococcus pettenkoferi* ^1^	1
*Corynebacterium variabile*	5	*Bacillus fordii*	1	*Streptomyces* sp.	1
*Kocuria rhizophila*	5	*Bacillus licheniformis*	1	*Weissella thailandensis*	1
*Psychrobacter* sp. ^2^	5	*Bacillus mycoides*	1	*Yersinia intermedia* ^2^	1

^1^ NASM. ^2^ Gram-negative.

**Table 5:** Prevalence and overview of species identified in newly infected quarters.

Microbiological Organism Newly Infected	# Quarters		# Quarters		# Quarters
*Staphylococcus* sp. ^1^	230	*Lactococcus* sp.	5	*Brevibacterium senegalense*	1
*Staphylococcus epidermidis* ^1^	220	*Streptococcus gallolyticus*	5	*Corynebacterium aurimucosum*	1
No_ID	180	*Acinetobacter lwoffii* ^2^	4	*Corynebacterium flavescens*	1
*Staphylococcus chromogenes* ^1^	161	*Brevibacterium luteolum*	4	*Corynebacterium glutamicum*	1
*Micrococcus luteus*	157	*Kocuria* sp.	4	*Corynebacterium tuberculostearicum*	1
*Staphylococcus haemolyticus* ^1^	127	*Lactococcus lactis*	4	*Enterococcus cecorum*	1
*Aerococcus viridans*	123	*Staphylococcus succinus* ^1^	4	*Enterococcus gallinarum*	1
*Corynebacterium bovis*	123	*Streptococcus canis*	4	*Enterococcus* sp.	1
*Staphylococcus aureus*	100	*Bacillus licheniformis*	3	*Enterococcus thailandicus*	1
*Corynebacterium amycolatum*	91	*Candida tropicalis*	3	*Fictibacillus* sp.	1
*Corynebacterium* sp.	90	*Lysinibacillus boronitolerans*	3	*Globicatella* sp.	1
*Streptococcus uberis*	74	*Moraxella* sp. ^2^	3	*Glutamicibacter mysorens*	1
*Staphylococcus simulans* ^1^	68	*Paracoccus* sp. ^2^	3	*Glutamicibacter protophormiae*	1
*Staphylococcus equorum* ^1^	46	*Staphylococcus hyicus* ^1^	3	*Klebsiella oxytoca* ^2^	1
*Staphylococcus xylosus* ^1^	45	*Weissella* sp.	3	*Kocuria carniphila*	1
*Lactococcus garvieae*	44	*Brachybacterium paraconglomeratum*	2	*Lysinibacillus* sp.	1
*Staphylococcus hominis* ^1^	37	*Candida* sp.	2	*Lysinibacillus sphaericus*	1
*Corynebacterium xerosis*	33	*Chryseobacterium oranimense* ^2^	2	*Micrococcus* sp.	1
*Mammaliicoccus sciuri* ^1^	28	*Citrobacter koseri* ^2^	2	*Paenibacillus woosongensis*	1
*Aerococcus* sp.	22	*Corynebacterium casei*	2	*Paenibacillus anaericanus*	1
*Enterococcus faecium*	22	*Corynebacterium confusum*	2	*Paracoccus denitrificans* ^2^	1
*Streptococcus dysgalactiae*	19	*Enterococcus pseudoavium*	2	*Proteus vulgaris* ^2^	1
*Staphylococcus capitis* ^1^	16	*Glutamicibacter arilaitensis*	2	*Pseudarthrobacter* sp.	1
*Enterococcus faecalis*	15	*Klebsiella pneumoniae* ^2^	2	*Pseudoglutamicibacter* sp.	1
*Corynebacterium variabile*	12	*Lysinibacillus xylanilyticus*	2	*Raoultella ornithinolytica* ^2^	1
*Staphylococcus rosti* ^1^	12	*Paenibacillus lactis*	2	*Rhodococcus pyridinivorans*	1
*Bacillus* sp.	11	*Pseudomonas thermotolerans* ^2^	2	*Serratia marcescens* ^2^	1
*Candida rugosa*	10	*Serratia liquefaciens* ^2^	2	*Solibacillus* sp.	1
*Corynebacterium frankenforstense*	10	*Staphylococcus gallinarum* ^1^	2	*Staphylococcus auricularis* ^1^	1
*Glutamicibacter* sp.	10	*Staphylococcus pettenkoferi* ^1^	2	*Staphylococcus caprae* ^1^	1
*Streptococcus* sp.	10	*Staphylococcus piscifermentans* ^1^	2	*Staphylococcus muscae* ^1^	1
*Psychrobacter pasteurii* ^2^	9	*Streptococcus agalactiae*	2	*Staphylococcus vitulinus* ^1^	1
*Candida krusei*	8	*Streptococcus dysgalactiae*	2	*Staphylococcus warneri* ^1^	1
*Corynebacterium stationis*	8	*Trueperella pyogenes*	2	*Streptococcus lutetiensis*	1
*Enterococcus saccharolyticus*	7	*Acinetobacter pittii* ^2^	1	*Streptococcus mitis*	1
*Psychrobacter* sp. ^2^	7	*Acinetobacter towneri* ^2^	1	*Streptococcus parasanguinis*	1
*Bacillus pumilus*	6	*Arthrobacter gandavensis*	1	*Streptococcus parauberis*	1
*Acinetobacter* sp. ^2^	5	*Bacillus cereus*	1	*Streptococcus salivarius*	1
*Brevibacterium* sp.	5	*Bacillus clausii*	1	*Wautersiella falsenii* ^2^	1
*Escherichia coli* ^2^	5	*Brachybacterium conglomeratum*	1		
*Kocuria rhizophila*	5	*Brachybacterium* sp.	1		

**^1^ NASM. ^2^ Gram-negative**.

## Data Availability

The datasets presented in this article are not readily available because they are part of an ongoing study that will be published.

## Supplementary Materials

The following supporting information can be downloaded at https://www.mdpi.com/article/10.3390/antibiotics15070683/s1. Figure S1: Enrolment and selection of newly and chronically infected cows and quarters in the calculation of the Simpson and Shannon–Wiener indices.
