# Supplementary material for: Variety of Culturable Bacteria Associated with Subclinical Mastitis in Dairy Cows, Based on the Simpson’s and Shannon–Wiener Diversity Indices
Source: Antibiotics (Basel). 2026 Jul 12;15(7):683. doi: 10.3390/antibiotics15070683 (PMC13405879; doi:10.3390/antibiotics15070683)

# Variety of culturable bacteria associated with subclinical mastitis in dairy cows, based on the Simpson's and Shannon-Wiener diversity indices.

**Michael Farre[1]* and Lærke Boye Astrup[1]**

[1] SEGES Innovation, Agro Food Park 15, 8200 Aarhus, Denmark

* Correspondence: mifa@seges.dk; Tel.: +4523835400

**Supplementary Figure S1.** Enrolment and selection of newly and chronically infected cows and quarters in the calculation of the Simpson and Shannon-Wiener indices.

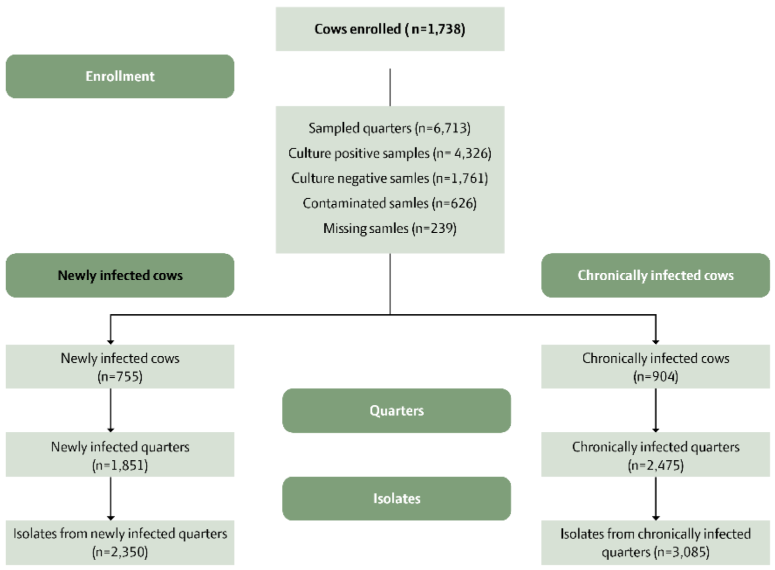

Supplement: Supplementary file 1 [file antibiotics-15-00683-s001.zip › antibiotics-4362233-supplementary.pdf]
